# Improving Imaging Quality of Real-time Fourier Single-pixel Imaging via Deep Learning

**DOI:** 10.3390/s19194190

**Published:** 2019-09-27

**Authors:** Saad Rizvi, Jie Cao, Kaiyu Zhang, Qun Hao

**Affiliations:** Key Laboratory of Biomimetic Robots and Systems, School of Optics and Photonics, Beijing Institute of Technology, Beijing 100081, China; srizvi@bit.edu.cn (S.R.); bitopzky@bit.edu.cn (K.Z.); qhao@bit.edu.cn (Q.H.)

**Keywords:** computational imaging, Fourier single-pixel imaging, deep learning

## Abstract

Fourier single pixel imaging (FSPI) is well known for reconstructing high quality images but only at the cost of long imaging time. For real-time applications, FSPI relies on under-sampled reconstructions, failing to provide high quality images. In order to improve imaging quality of real-time FSPI, a fast image reconstruction framework based on deep learning (DL) is proposed. More specifically, a deep convolutional autoencoder network with symmetric skip connection architecture for real time 96 × 96 imaging at very low sampling rates (5–8%) is employed. The network is trained on a large image set and is able to reconstruct diverse images unseen during training. The promising experimental results show that the proposed FSPI coupled with DL (termed DL-FSPI) outperforms conventional FSPI in terms of image quality at very low sampling rates.

## 1. Introduction

Single pixel imaging (SPI) [1] illuminates the target scene with structured patterns (random or basis) and records data over time (using a photodetector) to reconstruct spatial information about a target scene. Fourier single pixel imaging (FSPI) is a type of SPI which employs Fourier basis patterns to acquire the Fourier spectrum of a target scene [2]. SPI approaches like differential imaging [3], normalized SPI [4] and frequency-locked SPI [5,6] all aim at increasing measurement signal-to-noise ratio (SNR). However, FSPI achieves better measurement SNR [2] to produce high-quality images. Compared to a basis scan strategy like Hadamard single pixel imaging (HSI), FSPI is known to be more efficient and performs well on under-sampled image reconstruction [7]. In its simplest form, FSPI uses a digital micromirror device (DMD) to project phase-shifted sinusoidal illumination patterns onto a target scene and collects back-scattered light using an ordinary photodiode. By using inverse Fourier transform (IFT), a high-quality target image can be reconstructed. FSPI has gained popularity due to its low-cost design, imaging under background noise, and ability to operate over a long spectral range. Owing to these benefits, FSPI is transitioning from laboratory towards practical applications [8].

To reconstruct high quality images, FSPI requires a large number of measurements (equal to number of pixels in the target image) to acquire sufficient spatial information, which increases its imaging time. The imaging time of FSPI is characterized by data acquisition time and image reconstruction time. Since image reconstruction in FSPI is merely an inverse transform, its image reconstruction time is very low and does not pose a problem as in conventional SPI [9]. The data acquisition time for FSPI primarily depends on the modulation rate of a spatial light modulator (SLM). At present, commercially available DMDs (commonly used SLM) can operate maximum at ~22 kHz (fast FSPI [10]). Therefore, the imaging speed for FSPI is limited by the modulation rate of light modulator. In order to increase the imaging speed of FSPI, the only viable solution is to reduce its data acquisition time by capturing under-sampled images. For example, in practical applications, FSPI has been used for dynamic imaging [10,11] at ~10 fps with 22 kHz modulation rate. To achieve this frame-rate, the images were reconstructed at a 2% sampling rate which deteriorated the image quality. This confirms that image acquisition time offsets the true potential of FSPI for real-time imaging by compromising image quality.

To reduce the imaging time in SPI, compressive sensing (CS) methods have been applied [12,13,14]. CS techniques have proved to be quite efficient in recovering an image from fewer (compressive) measurements [15]. FSPI is similar to a CS method as it can reduce the number of measurements by selecting only a portion of the Fourier spectrum where most natural images are sparse. By acquiring under-sampled images using FSPI, there is a need for a reconstruction algorithm that can improve image quality from under-sampled measurements, supporting real-time FSPI. One promising option is to consider deep learning (DL) for image reconstruction in under-sampled FSPI.

Recent years have seen a surge of interest in employing DL for computational imaging. DL approaches can extract distinctive features from a large dataset and have been successfully employed for unsupervised learning in many applications. Particularly, DL has been applied in image dehazing [16], object classification through scattering media [17], hidden human identification [18], phase imaging [19], and single-pixel video [20]. DL also has the potential to significantly enhance the performance of FSPI for real-time applications. For FSPI, the most relevant deep neural network model is the denoising autoencoder [21]. It has been observed that an under-sampled image reconstructed using FSPI contains blurring artifacts. To remove these artifacts and reconstruct high-quality image, a deep convolutional autoencoder network (DCAN) was employed with symmetric skip connections that learn an end-to-end mapping between under-sampled images and ground truth. In this way, the model is trained to remove different types of noise and blurring artifacts inherent in FSPI reconstruction, and retain fine image details. 

This study demonstrates an imaging system that leverages the power of DL to reconstruct real-time high-quality 96 × 96 images. The proposed DCAN uses pairs of encoding and decoding layers connected by skip connections for improved image recovery and fast network convergence. The idea is to reduce acquisition time of FSPI by first acquiring under-sampled images at a 5–8% sampling rate, and then using our novel algorithm to reconstruct high-quality images with little computational cost to achieve higher frame rates. The proposed method can replace the conventional FSPI method for many real-time applications where a high-quality image is required at higher frame rates. Although work on increasing the frame rate of SPI has been done recently [22], FSPI still needs to make strides in this domain. Therefore, this work can provide guidelines for future application of DL in FSPI in this regard.

## 2. Principles and Methods

### 2.1. Fourier Single-Pixel Imaging

The imaging method of FSPI takes the Fourier transform as the basis. The idea is to capture the Fourier spectrum of a target scene by scanning the target scene with phase-shifting sinusoidal patterns and collect the back-scattered light using an ordinary photodiode. In this scheme, the method of 4-step phase shifting sinusoid was used to acquire the target image spectrum. This type of approach has proven to be robust against noise. The pattern for frequency pair *F* = (*f_x_, f_y_*) across image plane is generated using the expression [2]:

(1) $$P_{\phi }(x,y;f_{x},f_{y})=a+b\;\cos(2\pi f_{x}x+2\pi f_{y}y+\phi )$$

where *a* is the image intensity, and *b* is a contrast. The intensity back-scattered from the target scene integrated over the target can be given by:

(2) $$I_{\phi }(x,y;F)=\iint r(x,y)P_{\phi }(x,y;F)$$

where *r*(*x, y*) is the reflectivity distribution across the target plane. Considering the environment noise and random reflections near the scene, the total response encapsulated by the detector is written as [2]:

(3) $$R_{\phi }(F)=R_{n}+kI_{\phi }(x,y;F)$$

where *k* is associated with size of the detector [2], and *R_n_* is related to random light fluctuations around the detector. The following phase sequences are generated at a particular frequency to acquire the corresponding coefficients as: Pϕ=0→C0;Pϕ=π2→Cπ2;Pϕ=π→Cπ;Pϕ=3π2→C3π2. The phase shift between adjacent patterns is constant. By acquiring the response *R_ϕ_* for different phase values, a differential mechanism can be applied to cancel out noise, given by [2]:

(4) R0(F)−Rπ(F))+j(Rπ2(F)−R3π2(F))≈F{r(x,y)}.

Further applying the inverse Fourier transform (IFT), the image reconstruction is given by:

(5) F−1{(R0(F)−Rπ(F))+j(Rπ2(F)−R3π2(F))}≈r(x,y)

where *r*(*x, y*) is equal to the reconstructed image which is subsequently fed to the DCAN model. Through FSPI, the images are reconstructed at a very low sampling rate of 5–8%, allowing the DCAN to apply its learned model to improve image resolution and remove artifacts present in the under-sampled images. 

### 2.2. Deep Learning Based FSPI

**Figure 1:**
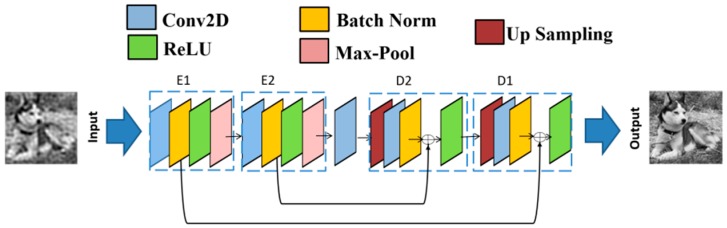
Deep convolutional autoencoder network (DCAN) architecture used in deep learning-Fourier single pixel imaging (DL-FSPI).

The proposed DCAN with symmetric encoding-decoding stages is shown in Figure 1. The network employs a convolutional layer (Conv2D) to extract features and remove corruptions using a set of trainable filters with a small receptive field. The encoding stages use 32 filters (5 × 5 × 1) and 64 filters (3 × 3 × 32). At the end of encoding stages, there is a single conv2D layer with 128 filters (3 × 3 × 64). The decoding stages use 64 filters (3 × 3 × 128) and 32 filters (3 × 3 × 64). The output is reconstructed using a single Conv2D filter (1 × 1 × 1). The network is initialized in an optimum state using Xavier initialization [23]. To accelerate the training process, every Conv2D layer is followed by a batch normalization (BN) layer [24]. The rectified linear unit (ReLU) nonlinear activation is used at every stage to avoid the vanishing gradient problem. The max-pooling layers are used to reduce dimensions and to provide transitional invariance. Conversely, the up-sampling layers restore the image resolution during decoding. To mitigate data over-fitting, *l2*-regularization (with same weights for all the layers) is used. During training, when the image data passes down the network pipeline, many smaller details are lost due to pooling and convolutional operations. To better reconstruct images, skip connections are used to traverse feature information between encoding and decoding stages which recovers important details and propagates gradients to deeper layers. The network architecture is carefully designed and fine-tuned to improve image quality with low computational time (for image reconstruction). After reconstructing under-sampled images via FSPI, the images are sent down the network pipeline for quality improvement. If *r*(*x, y*) is the target image, then the target captured by FSPI using under-sampled measurements is a corrupted version of the target image, given by:

(6) $$\tilde{r}\left(x,y\right)=H(r(x,y))+n$$

where *r*(*x,y*) is the clean image, $\tilde{r}\left(x,y\right)$ is the under-sampled image, *H* represents a degradation loss function, and *n* is the noise. Here, DL is chosen for solving the ill-posed inverse problem of estimating the original image from an under-sampled image. To achieve this, the network is trained to learn an end-to-end mapping from $\tilde{r}\left(x,y\right)\;\mathrm{to}\;r\left(x,y\right)$. For the reconstructed target $\hat{r}\left(x,y\right)$, the loss function that favors high peak signal-to-noise ratio (PSNR), for *m* training examples and parameterizing weights *Ɵ* can be expressed as:

(7) $$\min\;\mathrm{Loss}\;(\theta )\;=\;\frac{1}{m}\sum_{i=1}^{m}[\hat{r}(x,y)-r(x,y){]}^{2}$$

The network is fed with an under-sampled image reconstructed from FSPI explained in the above section. The reconstruction from under-sampled inputs through DCAN is depicted in Figure 1. To update network parameters and minimize loss, Adam optimization [25] was used with standard back propagation. The base learning rate (*lr* = 10^−4^) for all the layers was set to be the same.

The network was trained on STL-10 [26] DL dataset which contains 96 × 96 size images. All images were converted to gray scale and normalized before training. The training was performed on 10,000 unlabeled images. A test set (of 1000 images) was used to verify network performance during training, and a validation set (2000 images) was used to test the performance of the final model. Keras with TensorFlow was used to implement our model on an Intel i7 CPU (Integration Lenovo, Beijing, China) with 16 GB RAM. 

## 3. Results and Discussion

### 3.1. Simulations

**Figure 2:**
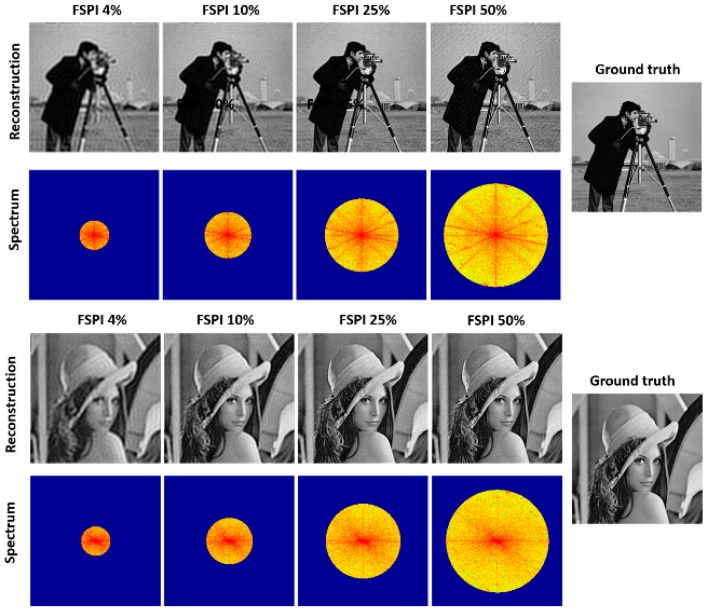
Fourier single pixel imaging (FSPI) reconstruction and amplitude spectrum for cameraman and Lena test images.

**Figure 3:**
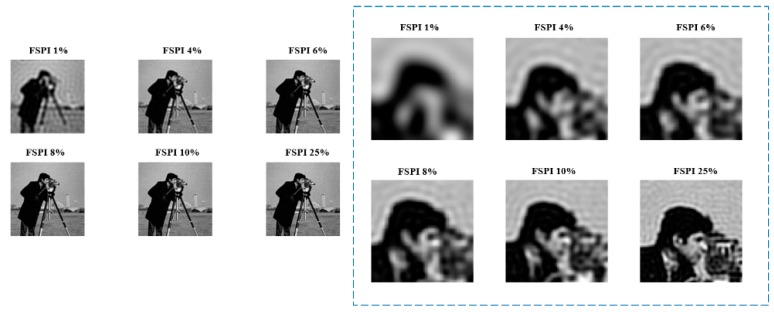
FSPI reconstruction for cameraman test image for 1–25% sampling rate. Right-side dotted box shows left side images zoomed in.

To observe how image quality deteriorates in FSPI under-sampled reconstruction, FSPI reconstruction was simulated for two test images i.e., Lena and cameraman, for different sampling ratios. The sampling rate (or ratio) ‘***S***’ (in percent) is the ratio between the *number of measurements* to *image size in pixels multiplied by 100*. The reconstruction results are shown in Figure 2. It can be seen that the image reconstruction quality for FSPI is very clear even for sampling ratios ***S*** ≤ 50%. However, for real-time applications, the FSPI reconstruction is usually based on ***S*** < 10% [10,11]. To observe image quality within 1–10% range, cameraman image is simulated for **S** = 1 to 10%, shown in Figure 3.

From Figure 3, it can be seen that the reconstructions for S between 1–10% has blurring artifacts present in the image. By qualitative comparison, it can be inferred that the clear target reconstruction is achieved at S = 25%. Therefore, for performance comparison, FSPI reconstruction at 25% sampling rate is set as the quality benchmark. This 25% benchmark for FSPI is more suitable with practical imaging, as the dynamics of reconstruction change for practical imaging. Since real-time FSPI uses lower sampling rates (S < 10%), it is necessary to develop an imaging framework that can produce high-quality images from the under-sampled images generated by FSPI.

**Figure 4:**
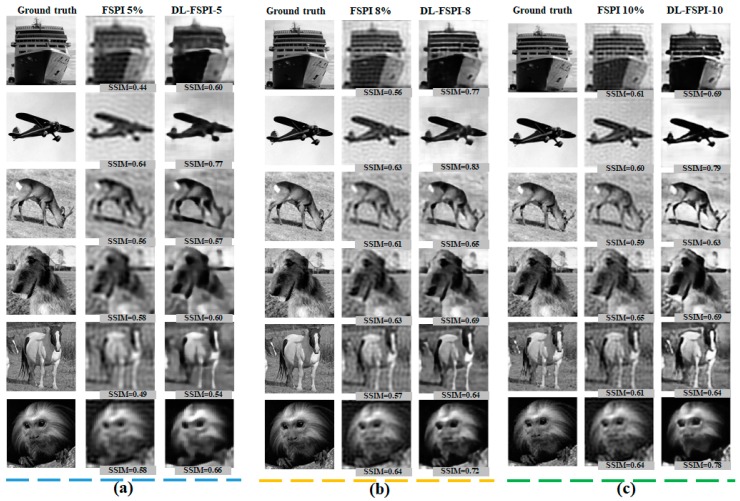
DL-FSPI reconstruction for the validation dataset for (**a**) 5%, (**b**) 8%, and (**c**) 10% sampling rates.

The proposed DL-FSPI framework was optimized by exhaustively testing it through numerical simulations. For training and testing, STL-10 dataset was used, which comprises of ten classes: Monkey, cat, dog, deer, car, truck, airplane, bird, horse, and ship. The DL-FSPI network was trained on training images reconstructed using conventional FSPI for 5%, 6%, 8%, and 10% sampling rates. For performance validation, 2000 images are kept aside as the validation dataset, which are not seen during training by the model. First, the performance of the proposed model was compared with conventional FSPI for different sampling rates using the validation dataset. For a qualitative and quantitative comparison between FSPI and DL-FSPI, the results of image reconstruction along with corresponding Structural SIMilarity (SSIM) [27] values are shown in Figure 4. 

**Figure 5:**
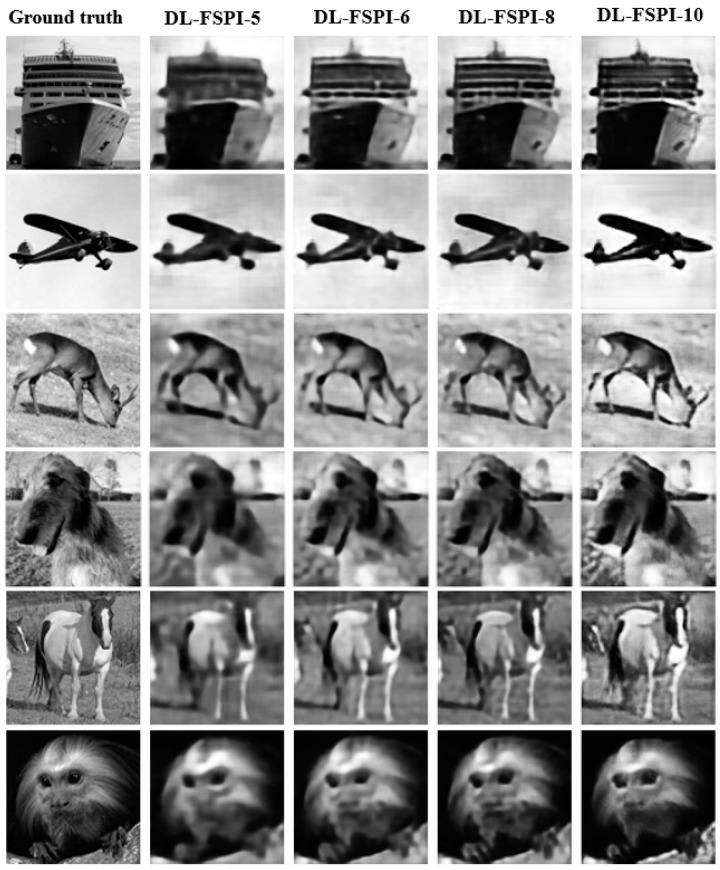
DL-FSPI reconstruction on validation dataset for 5%,6%, 8%, and 10% sampling rates.

It can be observed from Figure 4 that the proposed DL-FSPI can produce better quality sharp images compared to the corresponding FSPI method. The proposed DL-FSPI, after rigorous training on different types of under-sampled images and inherent FSPI artifacts, learns to reconstruct high-quality images from under-sampled inputs. Figure 5 shows the image reconstructions for different sampling rates by the DL-FSPI method. It can be observed from the figure that there exist a trade-off between sampling rate and image quality. For DL-FSPI-5 (imaging at ***S*** = 5%) which reconstructs images from 5% FSPI input, the reconstructed images have low quality. In this case, the model captures coarse details about the target scene due to blurring effects present in the under-sampled FSPI images. Therefore, in an attempt to achieve more compression, the image quality is lost. For sampling rates of 6%, 8%, and 10%, better image reconstruction quality can be observed. For the DL-FSPI-10 (imaging at ***S*** = 10%) model, the reconstruction results are the best amongst all other models, which is understandable because of the higher sampling rate. It can also be observed from Figure 5 that the image quality of DL-FSPI-6 (imaging at ***S*** = 6%) and DL-FSPI-8 (imaging at ***S*** = 8%) models is also comparable to that of DL-FSPI-10. Therefore, it can be concluded that up to 94% compression (using DL-FSPI-6) can be achieved without losing fine details in the image. However, for background sharpness and details, this study resorts to using DL-FSPI-8. 

**Figure 6:**
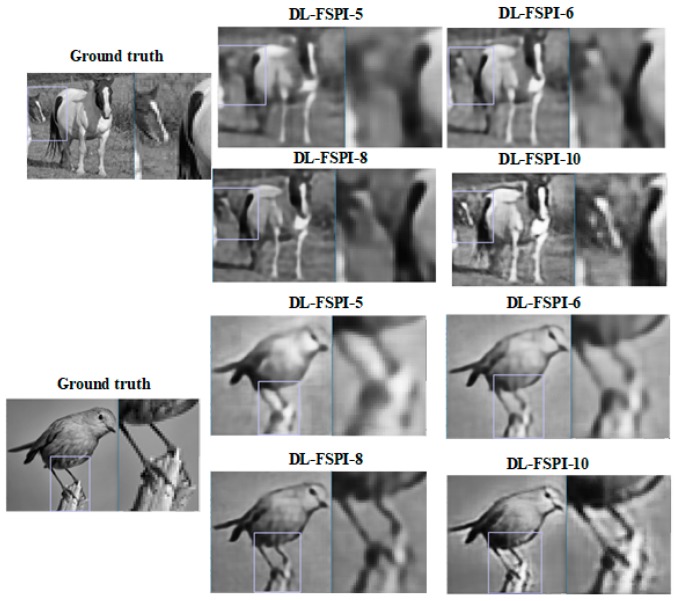
Background and low-level feature reconstruction for different DL-FSPI models.

**Figure 7:**
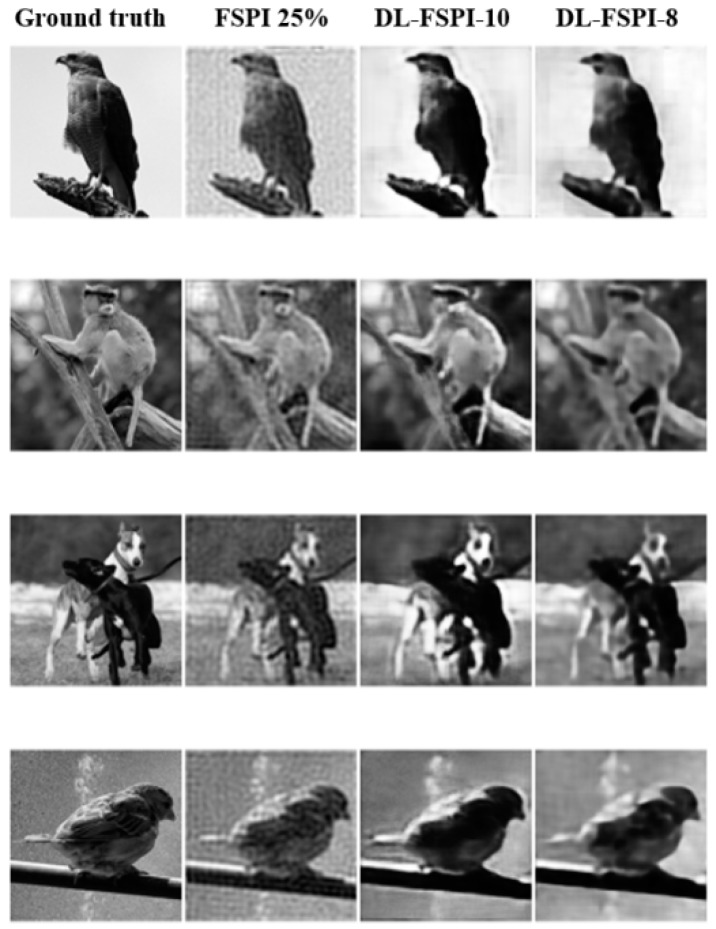
Image reconstruction quality comparison between FSPI and DL-FSPI.

Figure 6 shows target images reconstructed by the proposed DL-FSPI model at different sampling rates, with zoomed portions to inspect background or low-level details in the image. It can clearly be seen that both DL-FSPI-6 and DL-FSPI-8 models are able to reconstruct low-level features efficiently in the images. These fine details are further enhanced in the reconstruction by DL-FSPI-10. Conversely, DL-FSPI-5 is only able to recover coarse details in the image, with fine details appearing blurred in the zoomed portions of Figure 6. Furthermore, the image quality of the proposed method with the conventional FSPI (at 25%) method was compared. Figure 7 compares the image reconstruction quality of conventional FSPI (25%) with DL-FSPI (8% and 10%). It can be seen from this qualitative comparison that both DL-FSPI-8 and DL-FSPI-10 reconstruct high-quality images and the performance in most cases is better than conventional FSPI. The images reconstructed by DL-FSPI-10 are slightly brighter compared to DL-FSPI-8, but both models reconstruct fine details about the target clearly and accurately. Overall, the reconstruction by DL-FSPI methods is smooth with no artifacts.

**Figure 8:**
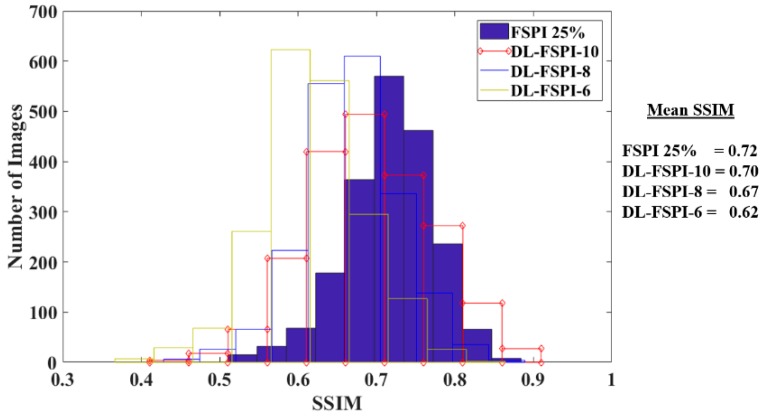
Histograms of SSIM for the validation dataset (2000 images) reconstructed using different methods.

For quantitative comparison, the performance of conventional FSPI (at 25%) was compared with the proposed DL-FSPI model using validation dataset. The reconstruction results are quantified using SSIM metric. Images from the validation dataset (2000 images) were reconstructed using conventional FSPI (***S*** = 25%) and DL-FSPI (***S*** = 6%, 8%, and 10%). The SSIM values of the reconstructions are plotted as histograms shown in Figure 8. The distribution from the histograms indicates that FSPI (25%) has slightly better reconstruction compared to DL-FSPI. However, the DL-FSPI method also outperforms FSPI (25%) for some images in the dataset. To quantify this performance comparison, the mean SSIM for the validation dataset for different methods is also presented in Figure 8. Both DL-FSPI-10 and DL-FSPI-8 compete well with 25% FSPI. Although the reconstruction quality of DL-FSPI method is similar to conventional FSPI (at 25%); the proposed method outperforms FSPI in terms of image reconstruction time. 

To quantify reconstruction time, different values of imaging time (physical experiment-based values) are presented for conventional FSPI and the proposed DL-FSPI in Table 1. The reconstruction time for conventional FSPI is the time taken by IFT, whereas for the proposed method this reconstruction time is the time taken by IFT pre-processing and the DL algorithm. From Table 1, it can be seen that the image acquisition time of FSPI is very long, whereas the proposed DL-FSPI method reconstructs similar quality images in a short time. This in turn affects the frame rate, which is critical for real-time applications. Therefore, our proposed method can generate more frames per second compared to conventional FSPI and can be used for real-time high-quality image reconstruction. 

**Figure 9:**
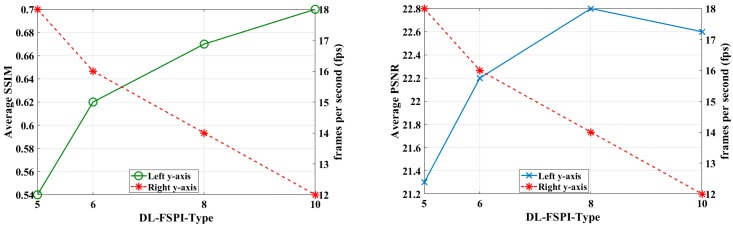
Quantitative comparison of DL-FSPI methods at different sampling rates.

The average PSNR and SSIM values were also computed for the reconstructed images in the validation dataset (2000 images) using different methods for quantitative comparison. Figure 9 shows the results for DL-FSPI-5, DL-FSPI-6, DL-FSPI-8, and DL-FSPI-10 methods. To select a particular method, there exists a trade-off between image quality and maximum achievable frame rate (*fps*). The trend in the graphs shows that as the image quality increases, the frame rate decreases. For rudimentary reconstruction, DL-FSPI-5 can be used to achieve higher frame rates. Whereas, for higher quality reconstruction, DL-FSPI-6, DL-FSPI-8 and DL-FSPI-10 (having higher frame rates compared to conventional FSPI) can be used.

### 3.2. Physical Experiments

**Figure 10:**
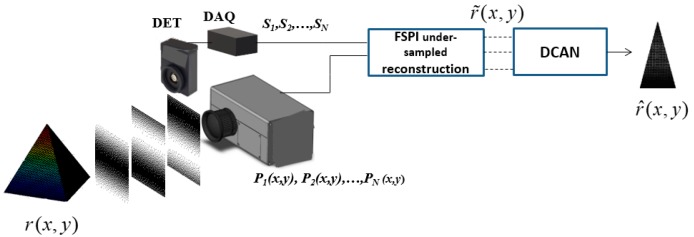
DL-FSPI experimental setup.

The experimental arrangement of DL-FSPI is shown in Figure 10. An integrated projection system was used to illuminate the scene with sinusoidal patterns. The projection system uses a light emitting diode (LED) operating at 450 nm (@30W) to illuminate the digital micromirror device (DMD) (TI DLP6500, Texas Instrument, Dallas, TX, USA). The light from the DMD is modulated and further projected onto the target using a projection lens. The scene to be captured is printed on a photograph paper for better quality reconstruction through FSPI, and is kept at a distance of 430 mm from the projector and photodetector. The light back-scattered from the scene is collimated onto the photodetector (18 mm^2^ active area, Thorlabs, Newton, NJ, USA) using an imaging lens (Computar H0514-MP2, 5 mm, Torrance, CA, USA). The intensity measurements from the photodetector were digitized using 16-bit data acquisition (DAQ) card (Gage CSEG8 sampling at 1.3 MS/s, Lockport, IL, USA). Customized software developed in LabVIEW was used to generate (and store) and project basis patterns as well as record intensity measurements from the photodetector. The software synchronously controls both DMD and photodetector. An Intel i7 CPU with 16 GB RAM was used for data processing.

The practical application of the proposed model was verified on diverse scenes (unseen during training) through two types of experiments.

(1) Experiment 1: In the first experiment, the under-sampled images were acquired (through FSPI) from the imaging setup, and then the network was trained on those images for reconstruction.
(2) Experiment 2: In the second experiment, the DL-FSPI model (DCAN block in Figure 10) trained on STL-10 dataset was applied directly onto the data from the imaging setup (under-sampled FSPI based images).

**Figure 11:**
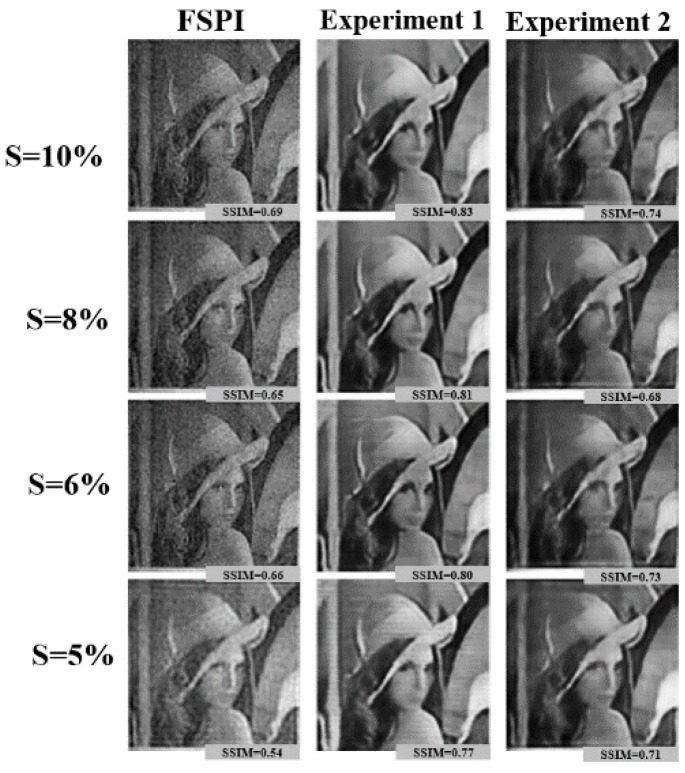
Comparison of reconstruction results for basic FPSI and DL-FSPI methods (experiment 1 and 2).

The results from the first experiment are shown in Figure 11. In this experiment, the images were taken from random datasets (Peppers, Lena, Dog etc.). The under-sampled FSPI reconstructions (5%, 6%, 8%, and 10%) were first acquired from the imaging setup and these were set aside as input images for training. The output label for training the network was set to be ground truth for the images under consideration. By training the network to learn an end-to-end mapping between the under-sampled FSPI images and ground truth counterparts, the network learns to remove noise present in the image from the imaging setup. Therefore, high-quality images can be reconstructed from the under-sampled inputs. The SSIM values corresponding to images in Figure 11 indicate that all DL-FSPI methods can produce high-quality image reconstructions. 

**Figure 12:**
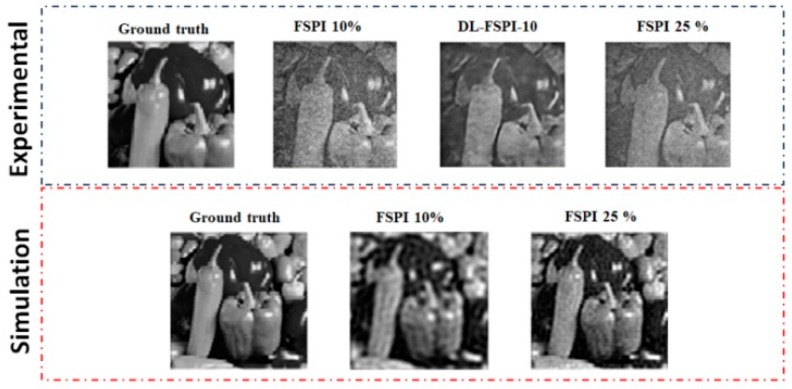
Reconstruction results of experiment-2 (also compared with simulation output).

The results from the second experiment are shown in Figure 11 and Figure 12. In this experiment, the DCAN model trained on STL-10 dataset was applied (through simulations) to reconstruct diverse target scenes. The difference between the simulations and experimental results are shown in Figure 12. The experimental results of Figure 12 show that the proposed model trained on the STL-10 dataset has enough knowledge of artifacts appearing in FSPI imaging that it easily removes them as seen in the DL-FSPI-10 image. It is important to note that as the sampling rate for FSPI increases to 25%, there still appears to be some fine-grained noise/artifacts in the image. Whereas, the DL-FSPI-10 image is sharp and the proposed algorithm removes all the artifacts and reconstructs a clear image. The SSIM values (compared with the ground truth pepper image) for Figure 12 experimental results are: FSPI 10% = 0.65, FSPI 25% = 0.62, DL-FSPI-10 = 0.71. 

## 4. Conclusions

This study focused on improving the efficiency of conventional FSPI, which fails to produce high-quality images in real-time. To shorten the imaging time and produce high-quality images, FSPI requires an efficient image recovery framework. This study proposed a novel image reconstruction framework for FSPI that leverages the power of DL to reconstruct real-time high-quality images from under-sampled low-quality FSPI images. The proposed DL-FSPI method employs a deep convolutional autoencoder network which uses symmetric pairs of encoding-decoding layers connected by skip connections for fast high-quality image reconstruction. Simulations and experiments validate the superiority of our model by comparing it with conventional FSPI method. The proposed method can replace the conventional FSPI method for many real-time applications where a high-quality image is required at higher frame rates. This work also provides guidelines for future application of DL in FSPI. Future investigations would involve characterizing the algorithm for very low S = 1–3%.

## Figures and Tables

**Table 1:** Experimntal Imaging time for FSPI and DL-FSPI.

Method	Acquisition Time (operating DMD at ~22 kHz)	Reconstruction Time (IFT or IFT+DL)	Imaging Time	Frames per Second (*fps*)
FSPI 25%	157 ms	9 ms	166 ms	6
DL-FSPI-10	63 ms	21 ms	84 ms	12
DL-FSPI-8	50 ms	21 ms	71 ms	14
DL-FSPI-6	38 ms	21 ms	59 ms	16
DL-FSPI-5	31 ms	21 ms	52 ms	18

## References

[1] Shapiro J. (2008). Computational ghost imaging. Phys. Rev. A.

[2] Zhang Z., Ma X., Zhong J. (2015). Single-pixel imaging by means of Fourier spectrum acquisition. Nat. Commun..

[3] Ferri F., Magatti D., Lugiato L., Gatti A. (2010). Differential ghost imaging. Phys. Rev. Lett..

[4] Sun B., Welsh S.S., Edgar M.P., Shapiro J.H., Padgett M.J. (2012). Normalized ghost imaging. Opt. Express.

[5] Sun B., Edgar M.P., Bowman R., Vittert L.E., Welsh S., Bowman A., Padgett M.J. (2013). 3D Computational Imaging with Single-Pixel Detectors. Science.

[6] Welsh S.S., Edgar M.P., Bowman R., Jonathan P., Sun B., Padgett M.J. (2013). Fast full-color computational imaging with single-pixel detectors. Opt. Express.

[7] Zhang Z., Wang X., Zheng G., Zhong J. (2017). Hadamard single-pixel imaging versus Fourier single-pixel imaging. Opt. Express.

[8] Peng J., Yao M., Cheng J., Zhang Z., Li S., Zheng G., Zhong J. (2018). Micro-tomography via single-pixel imaging. Opt. Express.

[9] He Y., Wang G., Dong G., Zhu S., Chen H., Zhang A., Xu Z. (2018). Ghost imaging based on deep learning. Sci. Rep..

[10] Zhang Z., Wang X., Zheng G., Zhong J. (2017). Fast Fourier single-pixel imaging via binary illumination. Sci. Rep..

[11] Huang J., Shi D., Yuan K., Hu S., Wang Y. (2018). Computational-weighted Fourier single-pixel imaging via binary illumination. Opt. Express.

[12] Katkovnik V., Astola J. (2012). Compressive sensing computational ghost imaging. J. Opt. Soc. Am. A.

[13] Katz O., Bromberg Y., Silberberg Y. (2009). Compressive ghost imaging. Appl. Phys. Lett..

[14] Candes E., Wakin M. (2008). An Introduction to Compressive Sampling. IEEE Signal Process. Mag..

[15] Donoho D.L. (2006). Compressed sensing. IEEE Trans. Inf. Theory.

[16] Cai B., Tao D., Xu X., Jia K., Qing C. (2016). DehazeNet: An End-to-End System for Single Image Haze Removal. IEEE Trans. Image Process..

[17] Satat G., Tancik M., Gupta O., Heshmat B., Raskar R. (2017). Object classification through scattering media with deep learning on time resolved measurement. Opt. Express.

[18] Caramazza P., Boccolini A., Buschek D., Hullin M., Higham C.F., Henderson R., Murray-Smith R., Faccio D. (2018). Neural network identification of people hidden from view with a single-pixel, single-photon detector. Sci. Rep..

[19] Sinha A., Lee J., Li S., Barbastathis G. (2017). Lensless computational imaging through deep learning. Optica.

[20] Higham C.F., Murray-Smith R., Padgett M.J., Edgar M.P. (2018). Deep learning for real-time single-pixel video. Sci. Rep..

[21] Vincent P., LaRochelle H., Bengio Y., Manzagol P.-A. Extracting and composing robust features with denoising autoencoders. Proceedings of the 25th International Conference on Machine Learning.

[22] Xu Z.-H., Chen W., Penuelas J., Padgett M., Sun M.-J. (2018). 1000 fps computational ghost imaging using LED-based structured illumination. Opt. Express.

[23] Glorot X., Bengio Y. Understanding the difficulty of training deep feedforward neural networks. Proceedings of the Thirteenth International Conference on Artificial Intelligence and Statistics.

[24] Ioffe S., Szegedy C. Batch normalization: Accelerating deep network training by reducing internal covariate shift. Proceedings of the International Conference on Machine Learning.

[25] Kingma D., Ba J. Adam: A method for stochastic optimization. Proceedings of the International Conference on Learning Representations.

[26] Coates A., Lee H., Ng A.Y. An analysis of single layer networks in unsupervised feature learning. Proceedings of the Artificial Intelligence and Statistics Conference.

[27] Wang Z., Bovik A.C., Sheikh H.R., Simoncelli E.P. (2004). Image quality assessment: From error visibility to structural similarity. IEEE Trans. Image Process..

